# Social media ratings of nursing homes associated with experience of care and “Nursing Home Compare” quality measures

**DOI:** 10.1186/s12913-019-4100-7

**Published:** 2019-04-27

**Authors:** Yue Li, Xueya Cai, Matthew Wang

**Affiliations:** 1 Department of Public Health Sciences, Division of Health Policy and Outcomes Research, University of Rochester Medical Center, 265 Crittenden Blvd., CU 420644, Rochester, NY 14642 USA; 2 Department of Biostatistics and Computational Biology, University of Rochester Medical Center, Rochester, USA; 3 Johns Hopkins University, Baltimore, USA

**Keywords:** Nursing home, 5-star rating, Social media, Experience of care

## Abstract

**Background:**

Social media platforms offer unique opportunities for patients and families to provide real-time feedback on their healthcare experiences. Consumer-generated social media ratings of hospitals tend to reflect the more subjective aspects of inpatient care experiences; however, evidence on nursing home care is extremely limited.

**Methods:**

We collected consumer-reported 5-star ratings of Maryland nursing homes posted from July 2015 to July 2017 on 4 popular social media or online review sites (Facebook, Yelp, Google Consumer Reviews, and Caring.com). We determined if the average score of social media ratings was associated with experience-of-care ratings derived from survey of family members or other responsible parties of nursing home residents, and with “Nursing Home Compare” (NHC) 5-star ratings and individual quality measures.

**Results:**

One hundred ninety-six out of 206 nursing homes in Maryland were reviewed on at least one site and thus had one or more star ratings posted. The overall ratings were 3.11 on average on these sites and 3.03 on the NHC website, with a Pearson correlation of 0.41 (*p* < 0.001) between the 2 sets of ratings. The correlations between the social media rating and survey-based experience-of-care ratings ranged from 0.40 to 0.60, and the correlations between the social media rating and individual NHC quality measures of citations, nurse staffing, and complaints were about 0.35 (in absolute values). The social media rating also predicted well NHC and experience-of-care measures after adjusting for nursing home covariates and market competition.

**Conclusions:**

The 5-star ratings collected from 4 social networking sites was correlated with and predictive of the NHC and survey-based experience-of-care measures for Maryland nursing homes.

## Background

Nearly 70% of individuals currently 65 years old will require long-term care during the remainder of their lives [1]. Each year, the nation’s 15,000 nursing homes provide residential post-acute and long-term care to over 3 million older and disabled Americans who are too frail to be supported in community-based settings [2]. Concerns exist that the quality of care in many nursing homes is less than adequate [3–5], and that patient outcomes and experience of care vary substantially over facilities [6–8]. Federal and state programs have been developed in the past several decades to address these quality deficits through stronger state regulations [9–11], as well as the national “Nursing Home Compare” report cards that publish key quality measures, such as nurse staffing and deficiency citations, to foster market competition and consumer choices of local facilities [12–14]. More recently, several states started to publicly report experience-of-care ratings of nursing homes which are derived from rigorously designed surveys of nursing home patients or their family members [8, 15–17]. Publications of these experience-of-care measures are intended to promote person-centered care in nursing homes that emphasizes collaborative care decision making, patient autonomy, and engagement of patients and family members [18].

In addition to these government published experience-of-care and quality measures, social media platforms, such as Facebook, Yelp, Google Reviews, and Twitter, offer unique opportunities for patients and families to provide real-time feedback on their experiences with individual healthcare providers [19]. Social media use has increased dramatically in the past decade with, for example, over 200 million unique Facebook users in the United States as of January 2018 [20], 170 million monthly visitors to Yelp in 2017 [21], and 245.5 million monthly visitors to Google in the U.S. in December 2017 [22]. Moreover, the use of social networking sites among adults 50 to 64 years increased from 33 to 51%, and usage among adults 65 and older tripled from 11 to 35%, during the period of 2010–2015 [23].

Emerging evidence suggests that consumer-generated social media ratings of hospitals tend to reflect the more subjective aspects of inpatient care experiences and may also be correlated with clinically-oriented quality measures [24–26]. Nevertheless, little is known if this is the case for nursing home care [27], and two recent studies analyzing on-line ratings of nursing homes available on Yelp [28] or Facebook [16] found no or minimal correlation with the 5-star ratings that the Centers for Medicare and Medicaid Services (CMS) developed based on deficiency of care, nurse staffing, and outcome measures.

This study collected on-line consumer ratings of Maryland nursing homes in the most recent two years (2015–17) from 4 popular social media or online review sites (Facebook, Yelp, Google Review, and Caring.com). We then determined if aggregated ratings from these crowdsourcing sites were associated with family-reported care experience scores, and with CMS’ “Nursing Home Compare” 5-star ratings and other quality measures.

## Methods

### Data sources

This study relied on data from 3 major sources: the archived Nursing Home Compare (NHC) data of 2017; the 2016 Maryland nursing home experience-of-care survey conducted by the Maryland Health Care Commission (MHCC); and on-line consumer ratings we collected from 4 popular social networking sites.

The NHC data are maintained and updated by the CMS and contain key nursing home characteristics such as facility name and address, nurse staffing, deficiency citations, consumer complaints filed against the facility, and 5-star ratings. The 5-star quality ratings were designed to simplify information for consumers by aggregating quality measures into a rating system of one to five stars, with more stars indicating better quality. The ratings are derived from 3 domains of quality: nurse staffing to resident ratios (for registered nurses [RNs] and all nursing staff including RNs, licensed practical nurses, and certified nursing assistants), deficiency citations (assigned during annual and complaint inspections), and clinical outcomes of residents based on Minimum Data Set assessments; an overall rating further aggregate the three domains [14].

The MHCC publishes on-line the nursing home experience-of-care rating scores annually based on mailed surveys of designated responsible parties (i.e. family members or legal guardians/representatives) of all long-term residents in Maryland [29]. This study followed the methodologies (survey design, samples, and survey methods) described in previous reports [15, 17, 29]. Briefly, the 2016 survey was conducted between March and June of 2016, and responses reflected family member evaluations of care provided from late 2015 to early 2016. All Maryland nursing homes serving long-term residents (*n* = 222 facilities) participated in this year’s survey and surveys were mailed to all responsible parties (*n* = 16,631) of their residents. Follow-up mails and phone calls were made to non-respondents. A total of 8356 completed surveys were received finally, resulting in an overall response rate of 53%. The survey asked 17 questions to assess 5 domains of resident care including (1) staff and administration, (2) care provided to residents, (3) food and meals, (4) autonomy and resident rights, and (5) physical aspects of the facility. The rating of each domain is the average of scores of all questions within the domain, and has a range between 1 (worst experience with care) and 4 (best experience with care). The survey also asked two additional questions about (1) overall experience with care in the nursing home on a rating from 1 (worst possible care) to 10 (best possible care) and (2) whether the respondent would recommend the nursing home to those who need nursing home care (yes/no).

Using the list of Maryland nursing homes published by the MHCC, we first conducted Google Maps search to obtain each nursing home’s Google Customer Reviews 5-star rating scores from past or existing patients/families, and to identify the web page of each facility as well. We then searched within each facility’s page for a link to the Facebook page and to the Yelp page of the nursing home. For nursing home websites that did not include a link to their Facebook or Yelp page, we searched Facebook and Yelp respectively for the facility’s official page, and confirmed this information using facility name and address. Finally, we identified all Maryland nursing homes from caring.com, an online reviews website specifically designed for customer search for and rating on professional senior care providers such as assisted living facilities, nursing homes, and hospices.

All the 4 crowdsourcing sites (Facebook, Yelp, Google Customer Reviews, and caring.com) allow customers to rate their experiences with healthcare providers using 1 (worst experience) to 5 (best experience) stars and post optional review texts [16, 25, 26, 28]. We collected all star-ratings posted on the 4 sites during the period of July 2015 to July 2017; no potentially identifiable information (e.g. reviewer ID or user name) was collected from these sites. We chose this period because it matches roughly the periods of data collection in the Maryland nursing home care experience survey (2016) and in the 2017 NHC quality measures (e.g. 5-star ratings largely derived from 2015 to 17 data).

### Analysis

We analyzed facility-level average score of 5-star ratings from the 4 social media or consumer review websites (hereafter referred to as social media ratings), experience-of-care ratings, NHC 5-star overall ratings, and individual NHC quality measures including annual number of deficiency citations, case mix adjusted hours per resident day for RNs and for all nurses, and number of complaints filed by consumers or caregivers against the facility during 2015–17 that resulted in a deficiency citation. The goal of these analyses was to determine how well the average score of social media ratings is correlated with or predictive of the NHC measures and the survey-based care experience ratings for Maryland nursing homes. We performed descriptive analyses and ran Pearson correlation analyses on all measures and scores from alternative sources.

We further fit separate multivariable linear regression models to test the association of the average social media rating score (independent variable) with each NHC measure or the overall or domain-specific experience-of-care rating (dependent variable). All regression models controlled for nursing home and county covariates including number of certified beds, total number of residents, profit status (for-profit or not), chain affiliation (yes/no), a case mix index calculated based on the Resource Utilization Groups classification system, percentages of Medicare residents in the nursing home, percentage of Medicaid residents, percentage of white residents, and a measure of market competition for nursing home care calculated from the county-level Herfindahl–Hirschmann index. In each model, the average social media rating, which ranged from 1 to 5 continuously, was categorized as < 2 stars, from 2 (inclusive) to 4 stars, and ≥ 4 stars, with the first group serving as the comparison group. We present adjusted NHC measures or experience-of-care ratings by social media rating group based on model predictions.

## Results

From the Maryland Health Care Commission 2016 report, we identified between 196 and 206 nursing homes that had scores for overall or domain-specific experience-of-care ratings (the scores for about 16–26 nursing homes were not published in the report due to low response rates). Of the maximum of 206 nursing homes, 196 were reviewed from July 2015 to July 2017 on at least one of the 4 social networking sites and thus had at least one star rating posted; specifically, 47 nursing homes (24%) had at least one star rating available on 1 social networking site only, 75 nursing homes (38%) on 2 sites only, 58 nursing homes (30%) on 3 sites only, and 16 nursing homes (8%) on all 4 sites searched. The numbers of nursing homes with at least one star rating posted on individual websites were 119 on Facebook, 51 on Yelp, 170 on Google Consumer Reviews, and 100 on caring.com, during the 2-year period.

**Fig. 1:**
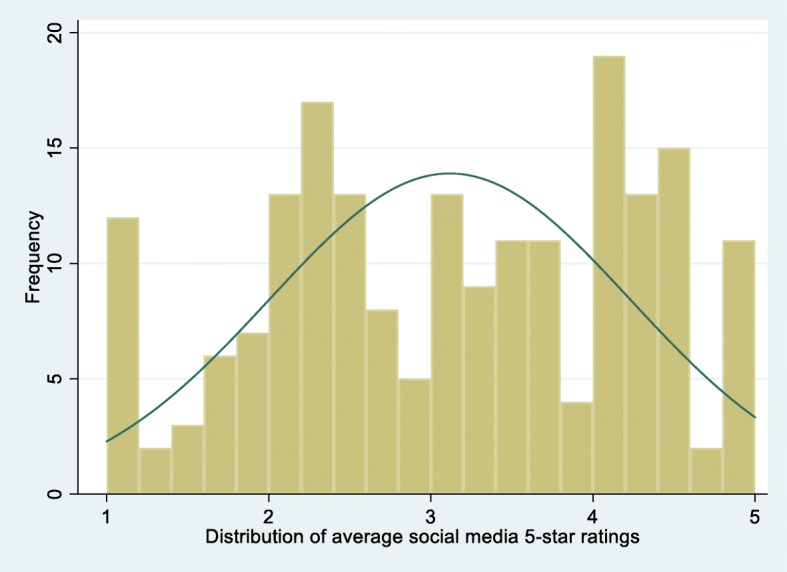
Average 5-star rating scores for Maryland nursing homes reported on 4 social media or online review sites (Facebook, Yelp, Google Consumer Reviews, and Caring.com) from July 2015 to July 2017

Figure 1 shows the distribution of the average rating scores for Maryland nursing homes reported on the 4 social media sites. Among the 196 nursing homes, the overall 5-star rating score was 3.11 on average (standard deviation [SD] = 1.11) on the 4 social networking sites and was 3.03 (SD = 1.37) on the “Nursing Home Compare” website (Table 1). According to the MHCC survey, the overall experience-of-care score was 8.21 on average (SD = 0.80) out of a range from 1 to 10, and 87% of surveyed family members or other responsible parties (SD = 12.1) would recommend the nursing home to others; the average scores for individual survey domains were around 3.5 (possible range 1–4) and varied over nursing homes. Other measures based on citations, nurse staffing, and complaints also varied over nursing homes in Maryland, as did other key facility characteristics such as size, resident census, and ownership types (Table 1).

**Table 1:** Descriptive statistics of Maryland nursing homes in 2016 (*n* = 196)*

	Mean ± SD or Prevalence (n)
Social media 5-star rating (1–5)	3.11 ± 1.11
Nursing Home Compare overall 5-star rating (1–5)	3.03 ± 1.37
Experience-of-care survey rating	
Overall rating (1–10)	8.21 ± 0.80
Percentage of recommendation (0–100)	86.6 ± 12.1
Staff & administration (1–4)	3.64 ± 0.18
Care provided to residents (1–4)	3.47 ± 0.21
Food & meals (1–4)	3.47 ± 0.23
Autonomy (1–4)	3.51 ± 0.27
Physical environment (1–4)	3.39 ± 0.25
Quality measure	
Total number of deficiency citations	12.38 ± 7.70
Adjusted registered nurse staffing (hours per resident day)	0.59 ± 0.25
Adjusted total nurse staffing (hours per resident day)	3.89 ± 0.79
Number of complaints	7.68 ± 8.92
Certified number of beds	127.5 ± 62.2
Total number of residents	112.4 ± 56.7
Occupancy rate	0.87 ± 0.10
For profit ownership	77.6% (152)
Chain affiliation	60.3% (118)
Case mix index	1.33 ± 0.14
Percentage of Medicare residents	21.20 ± 15.79
Percentage of Medicaid residents	56.98 ± 24.68
Percentage of white residents	66.10 ± 28.76
Competition for nursing home care	0.88 ± 0.15

*Our sample include 196 Maryland nursing homes with at least one social media rating, with Nursing Home Compare overall rating and quality measures, and with experience-of-care survey ratings

Table 2 shows that the correlation coefficient between the social media 5-star rating and that of the NHC was 0.41 (*p* < 0.001), indicating moderate correlation [30]. The correlations between the social media rating and MHCC’s experience-of-care ratings ranged from 0.40 to 0.60, which were slightly higher than the correlations between the NHC 5-star rating and MHCC’s experience-of-care ratings. The correlations between the social media rating and individual NHC quality measures of citations, staffing, and complaints were about 0.35 (in absolute values), which were somewhat lower than the correlations between the NHC 5-star rating and individual quality measures (*p* < 0.001 in all cases). In sensitivity analyses we limited the correlation analyses to nursing homes with at least 5 social media ratings posted (Appendix: Table 4; *n* = 130 nursing homes) or with at least 10 social media ratings posted (Appendix: Table 5; *n* = 72 nursing homes); the results were similar although the correlations between the social media rating and other ratings and quality measures were somewhat higher.

**Table 2:** Pearson correlation coefficients between social media 5-star rating, Nursing Home Compare (NHC) 5-star rating, experience-of-care survey ratings, and common quality measures for Maryland nursing homes

	Social media 5-star rating	NHC 5-star rating
Social media 5-star rating	–	0.41***
NHC 5-star rating	0.41***	–
Experience-of-care survey rating		
Overall rating	0.57***	0.53***
Percentage of recommendation	0.56***	0.46***
Staff & administration	0.46***	0.40***
Care provided to residents	0.49***	0.44***
Food & meals	0.40***	0.30***
Autonomy	0.47***	0.46***
Physical environment	0.53***	0.52***
Quality measures		
Number of deficiency citations	− 0.33***	− 0.49***
Adjusted registered nurse staffing	0.34***	0.39***
Adjusted total nurse staffing	0.36***	0.48***
Number of complaints	−0.35***	− 0.48***

***p < 0.001 in all cases

Results of multivariable regressions (Table 3) suggested that the social media rating predicted well other ratings and quality measures after adjusting for nursing home covariates and market competition. For example, compared to nursing homes with social media rating < 2 stars (average adjusted NHC 5-star rating 2.07), nursing homes with average social media rating of 2–4 stars had adjusted NHC 5-star rating of 2.80 (*p* < 0.05 for difference), and nursing homes with average social media rating ≥ 4 stars had adjusted NHC 5-star rating of 3.89 (*p* < 0.01). Similarly, compared to nursing homes with social media rating < 2 stars (adjusted number of complaints 13.38), nursing homes with average social media rating of 2–4 stars had adjusted number of complaints of 9.21 (*p* < 0.05), and nursing homes with average social media rating ≥ 4 stars had adjusted number of complaints of 2.67 (*p* < 0.01).

**Table 3:** Adjusted Nursing Home Compare 5-star rating, experience-of-care survey ratings, and quality measures for Maryland nursing homes, according to social media 5-star ratings

	Social media 5-star rating		
	< 2 stars (*n* = 32 nursing homes)	2–4 stars (*n* = 104 nursing homes)	≥4 stars (*n* = 60 nursing homes)
Adjusted NHC 5-star rating	2.07	2.80**	3.89***
Adjusted experience-of-care rating			
Overall rating (1–10)	7.64	8.05*	8.82***
Recommendation rate, %	77.6	85.2**	95.0***
Staff & administration (1–4)	3.55	3.61	3.76*
Care provided to residents (1–4)	3.34	3.43	3.61**
Food & meals (1–4)	3.36	3.42	3.61**
Autonomy (1–4)	3.40	3.45	3.69
Physical environment (1–4)	3.26	3.34	3.58**
Adjusted quality measures			
Number of deficiency citations	17.00	13.16	8.87**
Adjusted registered nurse staffing	0.49	0.57	0.73*
Adjusted total nurse staffing	3.56	3.79	4.31
Number of complaints	13.38	9.21**	2.67***

Note: prediction of each adjusted rating or quality measure was based on a linear regression model that had social media 5-star rating as the independent variable and adjusted for nursing home bed size, total number of residents, profit status, chain affiliation, case mix, percentages of Medicare and Medicaid residents, percentage of white residents, and market competition for nursing home care

*p < 0.10; **p < 0.05; and ***p < 0.01 when compared to the adjusted rating or quality measure in the reference group (social media rating < 2 stars)

## Discussion

This study found that the average score of 5-star ratings posted by consumers on 4 social media and consumer review websites (Facebook, Yelp, Google Consumer Reviews, and caring.com) was correlated moderately with the “Nursing Home Compare” 5-star rating and quality measures (Pearson correlations 0.30–0.40), and was correlated moderately to strongly [30] with the experience-of-care ratings derived from survey of family members (or other responsible parties) of Maryland nursing home residents (Pearson correlations 0.40–0.6). The social media rating score also predicted independently the “Nursing Home Compare” and experience-of-care scores after adjustment for common nursing home characteristics and market competition for nursing home care.

These findings are consistent with those of studies on the associations between social media ratings of hospital care and traditional hospital performance measures [24–26]. For example, Ranard and colleagues [25] reported that the average Yelp rating of hospitals had a Pearson correlation of 0.50 with an HCAHPS (Hospital Consumer Assessment of Healthcare Providers and Systems) overall experience-of-care score. Campbell and Li [26] focused on hospitals in New York State and found similar correlations of average Facebook consumer ratings with the overall HCAHPS score (Pearson correlation 0.54), as well as with HCAHPS scores for individual domains of care.

Two recent studies on nursing homes, however, reported that consumer ratings on Facebook [16] or Yelp [28] showed no or minimal correlations with the “Nursing Home Compare” and experience-of-care performance scores. The two studies searched and obtained consumer rating scores from each site (Facebook or Yelp), and their correlation analyses were limited to a small percentage (15–35%) of studied nursing homes that were reviewed on each site. Thus, the limited samples in these studies may bias their results. As one of the studies [28] and a commentary written by Dr. Bardach [27] pointed out, and as demonstrated in another recent study on English hospitals [31], aggregating data from alternative crowdsourcing sites might help improve the effort of identifying high-performing versus low-performing nursing homes.

This study improved on the data collection methods of previous studies by assembling rating scores from 4 popular social media or consumer review websites. The benefits of our improved data collection are that (1) it substantially reduced sample selection because the majority of nursing homes (i.e. 95% in this study) were identified as being reviewed on at least one site; and (2) it allowed us to select only consumer ratings posted in recent 2 years (2015–17) for analyses on these identified nursing homes, which presumably improved the accuracy of the estimated correlations and predictive abilities of social media ratings in regard to NHC and experience-of-care performance measures published around the same period of time. Of note, most social media or consumer review sites started their consumer rating system at least 5 to 10 years ago (e.g. 2013 on Facebook, and 2009 on caring.com) and the inclusion of posted ratings from all years would be another source of bias for estimated correlations.

Our study also showed that compared to NHC 5-star ratings, the aggregated social media ratings were slightly better correlated with MHCC’s experience-of-care ratings, but were somewhat less correlated with individual NHC quality measures (e.g. deficiency citations). This pattern may reflect the different aspects of nursing home care that individual measures tend to emphasize. Ratings on social media sites and in the MHCC survey may both largely reflect the perspectives of family members of residents and their experience with caregivers in the facility, and thus are expected to be well correlated. In contrast, the NHC 5-star ratings reflect an amalgamation of deficiency citations (state official evaluations of care problems during on-site inspections), nurse staffing patterns (self-reported by nursing homes), and clinical outcomes of residents (e.g. pressure ulcer rate), which were expected to be less well correlated with family reported ratings.

Social media ratings of nursing homes and other healthcare providers are real time, non-technical, and increasingly accepted by consumers and policy makers as a useful source of consumer feedback and voices [27]. Thus, they offer a new opportunity for informed consumer choice of high-quality nursing homes, market-driven quality improvement (e.g. through quality report cards), and person-centered care. These online consumer ratings are available for almost all nursing homes in the nation and tend to reflect family members’ and residents’ care experiences, which are not captured in the current “Nursing Home Compare” report cards but could be incorporated in these report cards in the future. Future research is also necessary to analyze the narrative reviews accompanying the posted ratings in order to identify specific topics of care experiences that underlie the ratings and to better inform future efforts of incorporating consumer perspectives into quality report cards.

We acknowledge potential limitations of this study. First, our analyses were limited to nursing homes in Maryland. Thus, results of this study should be generalized to nursing homes in other states with some caution. Second, our analyses were cross-sectional and therefore the estimated predictive abilities of social media ratings in multivariable analyses may be confounded by unmeasured nursing home and market characteristics. Future studies could collect the social media data over a longer period of time and determine longitudinally the associations and predictive abilities of social media ratings with respect to other traditional nursing home quality measures. In addition, although this study collected ratings posted on 4 alternative, popular social media or consumer rating sites, it is possible that a consumer gives the same rating score for a nursing home on alternative crowdsourcing sites which leads to an issue of multiple counting in calculating an average score. Although Facebook requires a log-in before reviews can be posted, other sites do not have this requirement and do allow for anonymity of reviewers. Thus, we had no way to identify multiple postings or possible manipulations of on-line reviews (although all sites, especially Yelp and caring.com implement their own filtering algorithm). Finally, due to the extended stay of many nursing home patients in a facility, their family members may be reluctant to give very negative reviews or very low on-line ratings for the facility. Thus, the overall social media ratings may somewhat over-estimate the actual care experiences of patients although this does not necessarily mask the comparative differences in rating scores across facilities, or reduce the correlations between social media ratings and other measures.

## Conclusion

In conclusion, the aggregate score of 5-star ratings collected from 4 social networking sites (Facebook, Yelp, Google Consumer Reviews, and caring.com) was correlated with and predictive of the “Nursing Home Compare” ratings and quality measures, and survey-based experience-of-care ratings published for Maryland nursing homes. Social media ratings of nursing homes may offer a new opportunity to incorporate consumer perspectives into existing quality report cards.

## Appendix

**Table 4:** Pearson correlation coefficients between social media 5-star rating, Nursing Home Compare (NHC) 5-star rating, experience-of-care survey ratings, and common quality measures for Maryland nursing homes with 5 or more social media 5-star ratings (n = 130 nursing homes)

		Social media 5-star rating
Social media 5-star rating	–	0.46***
NHC 5-star rating	0.46***	–
Experience-of-care survey rating		
Overall rating	0.65***	0.50***
Percentage of recommendation	0.63***	0.43***
Staff & administration	0.58***	0.38***
Care provided to residents	0.60***	0.39***
Food & meals	0.45***	0.27***
Autonomy	0.53***	0.43***
Physical environment	0.66***	0.47***
Quality measures		
Number of deficiency citations	−0.33***	−0.55***
Adjusted registered nurse staffing	0.38***	0.42***
Adjusted total nurse staffing	0.49***	0.47***
Number of complaints	−0.43***	−0.53***

****p* < 0.001 in all cases

**Table 5:** Pearson correlation coefficients between social media 5-star rating, Nursing Home Compare (NHC) 5-star rating, experience-of-care survey ratings, and common quality measures for Maryland nursing homes with 10 or more social media 5-star ratings (n = 72 nursing homes)

	Social media 5-star rating	NHC 5-star rating
Social media 5-star rating	–	0.40***
NHC 5-star rating	0.40***	–
Experience-of-care survey rating		
Overall rating	0.72***	0.51***
Percentage of recommendation	0.65***	0.42***
Staff & administration	0.66***	0.39***
Care provided to residents	0.65***	0.39***
Food & meals	0.57***	0.26**
Autonomy	0.61***	0.43***
Physical environment	0.67***	0.47***
Quality measures		
Number of deficiency citations	−0.35**	−0.61***
Adjusted registered nurse staffing	0.30*	0.32**
Adjusted total nurse staffing	0.48***	0.39***
Number of complaints	−0.50***	−0.61***

**p* < 0.05, ***p* < 0.01, ****p* < 0.001

## Abbreviations

CMS: Centers for Medicare and Medicaid Services
MHCC: Maryland Health Care Commission
NHC: Nursing Home Compare
RNs: registered nurses
